# Venetoclax + hypomethylating agents combined with dose-adjusted HAG for relapsed/refractory acute myeloid leukemia: Two case reports

**DOI:** 10.1097/MD.0000000000023265

**Published:** 2020-11-20

**Authors:** Hongxia Wang, Junjun Bai, Zhixin Pei, Bei Zhang, Junjie Wang, Xingli Lian, Qinglin Song

**Affiliations:** a Department of Hematology, Jiaozuo People's Hospital; b Pharmacy Intravennous Admixture Service, Second People's Hospital of Jiaozuo, Jiaozuo, China.

**Keywords:** hypomethylating agents, relapsed/refractory acute myeloid leukemia, venetoclax

## Abstract

**Rationale:**

Some acute myeloid leukemia (AML) patients are unresponsive to treatment or have remission followed by worsening of disease (known as relapsed/refractory AML [R/RAML]) after standardized treatment. The CAG/HAG regimen is not often used clinically because heterogenous patient responses, resistance, and hematopoietic bone marrow dysfunction have been reported with its use. We present 2 cases of R/RAML treated with a new combined therapy (venetoclax+ hypomethylating agents [HMAs]) in which the HAG dose was adjusted and effective in the first course of treatment.

**Patient characteristics:**

Case 1 involved a 23-year-old man who had suffered from AML for >4 years, and his FLT3 mutation status was positive at the initial diagnosis. After the first course of treatment with the standard-dose “Da” plan, the patient experienced complete remission. During the subsequent courses of treatment, the patient experienced 6 recurrences and was treated with the “ID Ara-C + MIT + sidaaniline” and “CAG + sidaaniline” regimens. However, the disease did not respond. Case 2 involved a 26-year-old man who received chemotherapy with the “Da,” “ID Ara-C,” “decitabine + half-dose CAG,” and “HAE” regimens. In this patients, remission could not be achieved. Reintroduction of the “ia” scheme also failed after treatment in our hospital.

**Diagnosis:**

Two patients were diagnosed with R/RAML.

**Interventions:**

The patient in case 2 received chemotherapy interventions, whereas the patient in case 1 refused to receive medical services at our hospital.

**Outcomes:**

The patient in case 1 was discharged after complete response treatment due to economic reasons and relapsed 2 months later. The patient ultimately died of infection and heart failure. The patient in case 2 is receiving a second cycle of chemotherapy.

**Lessons:**

We recommend the “venetoclax + HMAs combined with dose-adjusted CAH/HAG” regimen as an effective treatment for adult R/RAML.

## 1 Introduction

AML is a hematopoietic malignancy characterized by uncontrolled proliferation of clonal neoplastic cells and accumulation in the bone marrow of blasts with an impaired differentiation program. In the current clinical treatment, both cytarabine and anthracyclines at standard doses are adopted in induction therapy. Administration of medium or high doses of cytarabine and allogeneic hematopoietic stem cell transplantation are the most common forms of treatment in consolidation therapy.^[1–3]^ However, some patients are unresponsive or experience remission followed by worsening of disease (R/RAML) after standardized treatment.^[4]^ Because of patient heterogeneity, resistance, and hematopoietic dysfunction of bone marrow, the CAG/HAG regimen is not commonly used clinically.^[5]^ HMAs have dual antitumor effects, with a high dose used to kill tumor cells directly and a low dose used to activate antioncogenic mechanisms.^[6]^ Venetoclax, an inhibitor of Bcl-2, prevents cells from undergoing apoptosis, but resistance can occur.^[7]^ Therefore, we present 2 cases of R/RAML treated with a new combination therapy including venetoclax + HMAs with dose-adjusted HAG, which was shown to be effective in the first course of treatment.

## 2 Case presentation

Informed consent for publication was obtained from the patients. The drugs used in the new therapy for R/RAML used in these cases are approved by the Food and Drug Administration. All procedures were conducted in accordance with the guidelines of the Clinical Research Ethics Committee of Jiaozuo People's Hospital.

A 23-year-old man who initially presented with dizziness, fatigue, and rash sought medical service on February 8, 2015. An examination of bone marrow was conducted. The results showed active proliferation of the bone marrow and granulocytes, with a 70.4% content of myoblasts. In the cytoplasm of some cells, there were slender Auer bodies and azurophilic granules. Flow cytometry analysis demonstrated that abnormal CD45dim/CD117+ cells accounted for 72.07% of marrow cells, and their phenotype was CD34+, CD117+, CD38+, HLA-DR+, CD13+ and CD33+. Some of the cells expressed CD115, whereas CD16, CD14, CD11b, and CD11c were not expressed. The karyotype of the bone marrow cells was 46,XY, del(9)(q22) [9]/46, XY [11]. Genetic analyses indicated a positive FLT3-ITD mutation and CEBPA mutation, resulting in a diagnosis of AML-M2 (FLT3 + high risk). After 1 course of induction treatment with a standard dose of the “DA” regimen, the patient achieved complete remission, and minimal residual disease (MRD) was negative. Genetic analyses showed that the CEBPA mutation was present and the FLT3-ITD mutation was not present on August 4. The patient was treated with the “MA” plan for consolidation and strengthening, as well as lumbar puncture and sheath injection twice. No further treatment was given. In March 2016, he was hospitalized in our hospital due to epistaxis and skin purpura. Routine blood examination showed WBC 37.6 × 10^9^ cells/L, Hb 71 g/L, and platelets (PLT) 31 × 10^9^ cells/L. On March 18, reexamination of bone marrow showed active bone marrow hyperplasia, and the percentage of primordial granulocytes was 82.5%. The karyotype of the bone marrow cells was 46,XY [9]. All fusion genes were negative. Genetic analysis showed that NPM1 and CEBPA mutations were present, whereas other tested mutations were absent. These results suggested that the disease had relapsed. Standard-dose IA (IDA 20 mg D1–3, Ara-C 100 mg q12 h D1–6) combined with sorafenib 400 mg bid was given on March 21. On April 10, bone marrow examination showed a second complete remission, and MRD was negative. Genetic analysis showed that NPM1 C-terminal mutation was absent, but the CEBPA N-terminal mutation was present. The chromosome karyotype was 6,XY [14]. The patient had achieved a second complete response (CR). Early allogeneic hematopoietic stem cell transplantation was suggested, but the patient refused human leukocyte antigen (HLA) testing for financial reasons. Therefore, 4 courses of induction therapy were given: “ID Ara-C + IDA + sorafenib” (Ara-C 2.0 g q12 h D1–3, IDA 20 mg D1–3, sorafenib 400 mg bid) on April 10, 2016; “ID Ara-C + MIT + sorafenib” (Ara-C 1.5 g q12 h D1–3, MIT 20 mg D1 10 mg D2–3, sorafenib 400 mg bid) on June 10, 2016; “decitabine + CAG + MIT + sorafenib” (decitabine 25 mg D1–5, idarubicin 20 mg D1–4, cytarabine 35 mg q12 h D1–8, G-CSF 300 μg QD, mitoxantrone 3 mg D1–4, sorafenib 400 mg bid) on August 25, 2016; and “ID Ara-c + MIT” (Ara-c 2.0 g q12 h D1–3, MIT 10 mg D1–3) on November 1, 2016. During this period, bone marrow examination showed CR with MRD negativity. Lumbar puncture and sheath injection were performed 6 times. In January 2017, a specialized program was initiated to maintain the treatment. The patient came to the hospital again in July 2017. The routine blood examination showed WBC 50.6 × 10^9^ cells/L, Hb 120 g/L, and PLT 9 × 10^9^ cells/L, and 70% of the cells in the circulation were blasts. The white blood cell analysis results demonstrated active hyperplasia, and blasts accounted for 67.5% of cells. MRD analysis showed that abnormal myeloblasts accounted for 80.28% of cells. The karyotype was 46,XY [1]. Analysis of the mutated genes revealed that FLT3-ITD mutation was not present, WT1 mutation was present in 3.55% of cells, the CEBPa-ITD mutation was present, and the CEBPa-BZIP mutation was present. The patient was considered to have relapsed again. On July 14, the “IA” (IDA 20 mg D1–3, Ara-C 100 mg q12 h D1–7) chemotherapy regimen was given. On September 8, reexamination of the bone marrow showed a third CR, and MRD was negative. Allogeneic hematopoietic stem cell transplantation was recommended, but the patient refused it. On September 10, chemotherapy with “ID Ara-C + MIT” (Ara-C 1.5 g q12 h D1–4, MIT 10 mg D1–3) was given. In March 2018, the patient came to the hospital again. Routine blood examination showed WBC 134.2 × 10^9^ cells/L, Hb 104 g/L, and PLT 16 × 10^9^ cells/L, and blasts accounted for 86% of cells. The patient was considered to have relapsed again. “Ia” (IDA 20 mg D1–3, Ara-C 100 mg q12 h D1–7) chemotherapy was given on March 27. On May 4, reexamination of the bone marrow showed a fourth CR, and MRD was negative. “HD Ara-C” (Ara-C 4.5 g q12 h D1–3) chemotherapy was given on May 12. The bone marrow was reexamined on August 2, 2018. The results showed obvious hyperplasia, and blasts accounted for 25.5% of cells. MRD analysis showed that abnormal myeloid cells accounted for 21.26% of cells. Genetic analysis demonstrated that FLT3-ITD mutation was not present, NPM1 mutation was not present, WT1 mutation was present in 0.81% of cells (considered negative), the CEBPA-ITD mutation was present, and the CEBPa-BZIP mutation was present. On August 5, standard-dose IA (IDA 20 mg D1–3, Ara-C 100 mg q12 h D1–7) was given. On October 16, the myelogram showed that a fifth CR had been achieved, and the MRD analysis showed 0.48% abnormal myeloid cells. Lumbar puncture suggested central nervous system leukemia, and repeated lumbar puncture, sheath injection, and craniocerebral radiotherapy were carried out. Hypoesthesia of both lower limbs and dysesthesia upon defecation appeared. The MRI showed abnormalities in ribs 3 to 11, consistent with changes in the spine caused by hematological disease. The patient was diagnosed to have extramedullary relapse of AML. On November 5, 2018, and January 15, 2019, the “ID Ara-C + MIT + sidaaniline” (Ara-C 2.0 g q12 h D1–3, MIT 20 mg D1, 10 mg D2–3, sidaaniline 30 mg qid PO) and “CAG + sidaaniline” (arubicin 20 mg D1–4 D11–14, cytarabine 20 mg q12 h D1–14, G-CSF 300 μg QD, sidaaniline 30 mg qid PO) regimens were given. On February 13, reexamination showed that osteopoiesis was active, with 16% of the original cells. The MRD result was 21.25%, and WT1 mutation was found in 15.38% of cells. On February 18, 2019, “venetoclax + aza” (venetoclax 100 mg/day increased daily to 400 mg/daily for maintenance, azacytidine 100 mg D3–9) chemotherapy was given. On March 9, the “hag” (homoharringtonine 2 mg × 7 days, cytarabine 100 mg q12 h × 7 days, GSF) scheme was added. The bone marrow showed active hyperplasia, 38% of original components, and the MRD result was 46.38%. On April 12, reexamination of the bone marrow showed CR and MRD negativity. No leukemic cells were found in the lumbar puncture on April 13. The patient refused to continue chemotherapy after CR treatment due to financial difficulties and relapsed 2 months after the CR. Pulmonary infection was found at admission, and the patient ultimately died of infection plus heart failure. The progression-free survival was 2 months, and the overall survival time was 47 months.

### 2.1 Case 2

A 26-year-old male with headache and gingival swelling sought medical service at our hospital in February 2019. On February 1, the bone marrow examination indicated extreme hyperplasia. The proportion of monocytes was significantly increased, with monocytes accounting for 67% of cells (57.2% of cells were monocyte progenitors). The patient was diagnosed with AML-M5. Following induction chemotherapy with the “DA” regimen, the disease did not respond. The patient was discharged from the hospital after chemotherapy with the “ID Ara-C” regimen. In June 2019, he returned to the hospital for perianal abscess and cervical lymphadenopathy. Routine blood examination showed WBC count 4.86 × 10^9^ cells/L, Hb 83 g/L, and PLT 20 × 10^9^ cells/L. On June 15, the bone marrow was reexamined. The number of blasts increased significantly, with blasts accounting for 92.5% of cells. These blasts were different sizes, rich in the cytoplasm, blue staining, and scattered purple-red particles. Flow cytometry showed that the immature cell group accounted for 95.68% of cells. Most of the cells expressed CD33, CD64, and CMPO; some of them expressed CD123, CD117, and CD38; and a few cells expressed CD13 and HLA-DR. AML recurrence was considered according to the pathological findings of the bone marrow biopsy. Chemotherapy was stopped on the second day because the patient did not tolerate it. In August 2019, the patient came to our hospital due to intermittent high fever. Routine blood examination showed WBC 9.94 × 10^9^ cells/L, Hb 71 g/L, and PLT 7 × 10^9^ cells/L, and blasts accounted for 86% of cells. On August 4, the bone marrow examination showed active proliferation, with blasts representing 24% of cells, immature precursors representing 72% of cells, and 100% of cells with granules. The flow cytometry restuls showed that abnormal myeloblasts accounted for 95.7% of cells, and these cells had expression of CMPO, CD33, CD64, and CD4 and partial expression of CD117, HLA-DR, and CD11b. The karyotype was 48,XY, + I (10) (Q10), t(11,19)(q23; P13), +13 [20]. Analysis for the presence of 43 fusion genes demonstrated mll-ell in 23.88% of cells (+) and NRAS, IKZF1, and ABCD1 mutations. According to these results, the patient was diagnosed with refractory AML-M5 (MLL-ELL+, NRAS+, IKZF1+). On August 13, standard-dosef IA (IDA 20 mg D1–2, 10 mg D3, Ara-C 100 mg q12 h D1–7) was given. The patient suffered from DIC during the course of chemotherapy, which was relieved by symptomatic treatment, including plasma infusion and cold precipitation. On September 3, reexamination showed that the bone marrow was proliferative and active, with 19% blasts, 34.5% precursors, and 39.33% abnormal myeloid cells. On September 8, the “venetoclax + aza” regimen was given. On September 26, the reexamination of bone marrow showed that the hyperplasia of bone marrow was decreased, blasts accounted for 1% of cells, and precursors accounted for 10.4% of cells. MRD analysis showed that abnormal myeloid blasts accounted for 9.7% of cells. Starting September 26, we added the “hag” (homoharringtonine 2 mg × 5 days, cytarabine 100 mg q12 h × 7 days, GSF) regimen. Reexamination showed a CR and MRD negativity. Allogeneic hematopoietic stem cell transplantation was suggested. At present, the patient is still in a CR state and the second cycle of “venetoclax + aza + hag” chemotherapy is being administered. Progression-free survival and overall survival durations have not been calculated.

## 3 Discussion

According to guidelines approved by NCCN, AML is characterized by the presence of more than 5% leukemia cells in peripheral blood or bone marrow after complete remission.^[8]^ The majority of scholars consider a patient to have R/RAML if a complete remission does not occur after 2 courses of induction chemotherapy, and when this occurs, the treatment plan needs to be adjusted.^[9–11]^ Because of the high failure rate of reinduction treatment after relapse of AML, R/RAML is common in the clinic. R/RAML has always been difficult to treat and a focus of AML research, and it is characterized by a short survival period and progressive complications. Due to chemotherapy resistance, the remission rate is low, and the incidence of adverse reactions is high during treatment.^[12]^ At present, there are many clinical research applications, such as flag, CAG^[13]^ and FLAG-IDA,^[14,15]^ flairg, and HAA. However, the CR rate is only 30% to 40%. Allogeneic hematopoietic stem cell transplantation is one of the most effective treatment methods. Therefore, it might be the only choice for curing R/RAML patients after the appropriate reinduction scheme achieves CR, and such a strategy could provide improve the disease-free survival rate and prolong the survival period.

Since the CAG/HAG regimen was first employed by Japanese scholars,^[16]^ it has been widely used and improved by Chinese hematologists.^[16,17]^ Given that these patients are often so difficult to treat, bone marrow images are reexamined often during treatment to identify response. Modifications to the timing of chemotherapy drugs have been made, and the remission rate of R/RAML has improved, but these improvements are only seen when the patients’ bone marrow also improves. Hag, which features small doses of homoharringtonine and cytarabine, is commonly used in the treatment of AML, and it is well tolerated and safe in patients. Homoharringtonine, which does not show cross-tolerance with cytarabine, is a biological ester with anticancer effects. Homoharringtonine can inhibit the synthesis of protein in eukaryotes, depolymerize polyribosomes, interfere with the function of ribosomes, and control the synthesis of DNA in cells. It may induce leukemic cells to undergo apoptosis and inhibit the growth of leukemic cells by inhibiting the JAK/STAT, PI3K, and WNT as well as other signaling pathways.^[18]^ Cytarabine is a pyrimidine antimetabolic drug mainly acting in S phase^[18]^ that can interfere with cell proliferation and induce cell apoptosis by competitively inhibiting DNA aggregation and synthesis. Granulocyte colony-stimulating factor can make cells in G0 phase enter Gs phase and regulate sensitivity to specific drugs. Additionally, it can increase the level of cytarabine triphosphate, an active metabolite of cytarabine, thus enhancing the toxicity of cytarabine against AML progenitor cells and immature cells.^[19]^ In addition, granulocyte colony-stimulating factor can be released into peripheral blood, promote granulocyte maturation, and decrease the duration of bone marrow suppression.

With further study of the pathogenesis of leukemia, more molecular changes are being recognized,^[20]^ which directly affects the prognosis of patients. Epigenetic changes have attracted increasing attention. DNA demethylating drugs (HMAs) and HDACi are a major research focus in the treatment of AML.^[21]^ DNA methylation is the most common molecular change in AML. Clinical studies have found that regardless of the presence of abnormal gene expression, there is abnormal DNA methylation, and it is related to prognosis.^[22]^ DNA methylation plays an important role in the differentiation of hematopoietic stem progenitor cells, and CpG island methylation and histone methylation in the gene promoter regions regulate gene expression. Low methylation can lead to chromosome instability and abnormal activation of genes. High methylation can lead to gene silencing,^[23]^ which is usually reversible. Aberrant methylation also provides a new therapeutic target for AML. In recent years, combinations of HMAs and induction chemotherapy agents have been a new approach to treat AML patients. The combination of HMAs and induction chemotherapy agents can improve the cytotoxic effect of cytarabine and further improve its efficacy. Azacytidine (AZA) is a cytidine analog. In 2008, the European Drug Administration approved azacytidine for the treatment of high-risk MDS and AML (bone marrow blasts 20% - 30%).^[24]^ AZA mainly produces antitumor activity through two mechanisms: 1, by binding with DNA and RNA, it produces cytotoxic and specific cell cycle inhibition; and 2, by binding with DNA methyltransferases, it inhibits the methylation of newly synthesized DNA to restore the normal growth and differentiation of hematopoietic stem cells.^[24,25]^ Azacytidine is the only demethylating drug clinically proven to improve overall survival in AML/MDS patients.^[26]^

In recent years, new drugs targeting AML have been launched successfully. Among various AML targeted therapies, a new strategy is to directly stimulate mitochondrial apoptosis of cancer cells.^[27]^ This pathway is regulated by the Bcl-2 protein family. Overexpression of Bcl-2 causes tumor cell apoptosis and produces resistance to a variety of antitumor drugs.^[28]^ The occurrence of tumor neovascularization can be controlled by inhibiting the expression of Bcl-2, which results in the inhibition of tumor metastasis. Venetoclax is an oral and powerful selective inhibitor of Bcl-2. Venetoclax can activate the endogenous mitochondrial apoptosis pathway, promote the release of inflammatory factors from immune cells, and cause tumor cell apoptosis.^[29]^ Bcl-2 is highly expressed in AML progenitor cells, and Bcl-2 inhibitors may enhance the sensitivity of AML cells to demethylating drugs.^[30]^ It has been confirmed in vitro that venetoclax and AZA have a synergistic antileukemia effect.^[31]^ Mcl-1 is a protein from the Bcl-2 protein family and plays a key role in the survival of AML cells. It may contribute to venetoclax resistance. Studies have shown that AZA can significantly reduce the numbers of Mcl-1 in cells. All of the above findings provide a theoretical basis for the combination of the 2 drugs.^[32,33]^ DiNardo et al reported a clinical trial of the combination of the 2 drugs in the treatment of elderly AML patients of age ≥65 years. The results showed that the total response rate was 71%, higher than the total response rate to standard AML chemotherapy (50%–60%).^[34]^ Aldoss et al reported that 33 cases of R/RAML were treated with venetoclax combined with decitabine or azacytidine. Ten of them obtained CR (30%), and 3 of them obtained CRI (21%).^[35,36]^ At the 56th Annual ASH Conference, the Abbvie company presented phase II clinical data from a multicenter, nonblinded clinical trial involving 32 patients with AML. Thirty of them (93.8%) were thought to have relapsed and refractory AML. At the initial evaluation (at the end of the 4th week), 6 of 32 patients (19%) had a reduction in bone marrow blasts of >50%. Five patients (15.5%) had complete remission, but 4 of them relapsed. Among 4 patients with CRI, 1 patient achieved complete remission in the 20th week, and no acute tumor lysis syndrome occurred in the monitoring.

Venetoclax, AZA, and HAG have some anti-AML effects, but these are limited when the agents are used alone. They have synergistic effects on directly killing and regulating the bone marrow microenvironment. In this report, 2 patients with R/RAML achieved CR after a course of treatment with a “venetoclax + HMAs combined with dose-adjusted CAH/HAG” regimen, which provided the opportunity for bone marrow transplantation. The main adverse reactions were grade III and IV myelosuppression, nausea, oral ulcers, and constipation. The incidence of side effects was similar to that seen with conventional chemotherapy.

In summary, “venetoclax + HMAs combined with dose-adjusted CAH/HAG” is an effective method for adult R/RAML that has clinical value. Due to the limited number of cases and short observation times in this study, more cases featuring treatment with this scheme in adult R/RAML need to be investigated.

## Author contributions

**Conceptualization:** Hongxia Wang, Qinglin Song.

**Data curation:** Hongxia Wang, Junjun Bai, Qinglin Song.

**Formal analysis:** Hongxia Wang, Zhixin Pei.

**Funding acquisition:** Qinglin Song.

**Investigation:** Zhixin Pei, Bei Zhang, Junjie Wang, Xingli Lian.

**Methodology:** Junjun Bai, Zhixin Pei, Junjie Wang, Qinglin Song.

**Project administration:** Junjun Bai, Zhixin Pei, Junjie Wang, Qinglin Song.

**Resources:** Bei Zhang, Junjie Wang, Xingli Lian.

**Software:** Bei Zhang, Xingli Lian.

**Supervision:** Qinglin Song.

**Validation:** Bei Zhang, Xingli Lian, Qinglin Song.

**Visualization:** Bei Zhang.

**Writing – original draft:** Hongxia Wang, Qinglin Song.

**Writing – review & editing:** Hongxia Wang.
